# Qiliqiangxin Improves Cardiac Glucose Metabolism After Myocardial Infarction Through a HIF‐1α/MIF/AMPK Axis

**DOI:** 10.1111/jcmm.71238

**Published:** 2026-07-20

**Authors:** Zimu Wang, Zhonglei Xie, Hanqing Zhao, Chaofu Li, Yanyan Wang, Yu Song, Ya'nan Qu, Jingfeng Wang, Jingmin Zhou

**Affiliations:** ^1^ Department of Cardiology, Zhongshan Hospital, Fudan University Shanghai Institute of Cardiovascular Diseases Shanghai China; ^2^ Institutes of Biomedical Sciences Fudan University Shanghai China; ^3^ Department of Rehabilitation Medicine Xuzhou Center Hospital Jiangsu Province China; ^4^ Bioengineering College of Chongqing University Chongqing University Central Hospital (Chongqing Emergency Medical Center) Chongqing China; ^5^ Department of Cardiology Shanghai Geriatric Medical Center Shanghai China

**Keywords:** glucose metabolism, myocardial infarction, Qiliqiangxin *(QL)*

## Abstract

Cardiac glucose metabolism is critically involved in the pathophysiology of myocardial infarction. This study investigated whether Qiliqiangxin (*QL*), a traditional Chinese medicine, improves myocardial glucose uptake and aerobic oxidation after myocardial infarction (MI) and explored the underlying mechanisms. *QL* treatment significantly increased LVEF, enhanced myocardial glucose uptake, reduced fibrosis, apoptosis, ROS production and elevated ATP generation along with activity of pyruvate dehydrogenase (PDH) and citrate synthase (CS). These effects were abolished by HIF‐1α‐shRNA transduction. *QL* also upregulated the expression of HIF‐1α, MIF, p‐AMPKα, GLUT‐4, hexokinase 2 (HK2), PDH and CS, which were similarly attenuated by HIF‐1α knockdown. In conclusion, our study demonstrated that *QL* enhances myocardial glucose uptake and aerobic oxidation in a murine model of post‐MI heart failure. Genetic inhibition of HIF‐1α reverses these beneficial effects, suggesting the involvement of the HIF‐1α/MIF/AMPK signalling axis.

## 1 Introduction

Myocardial infarction (MI) remains a leading cause of global morbidity and mortality. While early reperfusion and optimal pharmacotherapy have significantly reduced acute mortality, the subsequent rise in the prevalence and mortality from post‐MI left ventricular (LV) remodelling and heart failure poses a persistent clinical challenge [1]. The heart, as the organ with the highest energy demand, is critically dependent on efficient energy metabolism. Impaired cardiac energetics is a major contributor to heart failure progression. Cardiac energy metabolism comprises three key components: substrate utilization, oxidative phosphorylation, and ATP transfer/utilization. Prior studies suggest that enhancing myocardial energy supply can be beneficial; for instance, intracoronary pyruvate infusion improves short‐term cardiac function, and increased glucose utilization is associated with improved LV function [2, 3]. Conversely, impaired oxidative phosphorylation compromises cardiac function by failing to meet cardiomyocyte ATP demands. Glucose aerobic oxidation, a crucial energy‐yielding pathway, is regulated by oxygen availability and key enzymes such as pyruvate dehydrogenase (PDH) and citrate synthase (CS). Targeted manipulation of these metabolic regulators may therefore improve cardiac energetics and the prognosis of ischemic heart failure.

Qiliqiangxin (*QL*), a traditional Chinese medicine compound derived from 11 herbs, has demonstrated efficacy and safety in treating chronic heart failure secondary to ischemic injury or pressure overload [4, 5]. Emerging evidence indicates that QL confers cardioprotective benefits, potentially through regulating glucose metabolism and enhancing mitochondrial biogenesis [6, 7]. However, it remains unclear whether QL can specifically promote glucose aerobic oxidation in the context of MI‐induced heart failure, and the precise mechanisms underlying its metabolic effects are not fully defined.

Therefore, the present study aimed to investigate whether *QL* enhances myocardial glucose uptake and aerobic oxidation in a murine post‐MI heart failure model and to elucidate the related mechanisms.

## 2 Methods

### 2.1 Construction of Adeno‐Associated Virus Serotype 9 (AAV9)

The recombinant adeno‐associated virus serotype 9 (AAV9) vectors were constructed and supplied by Cyagen Technologies (Guangzhou, China). Briefly short hairpin RNA (shRNA) targeting hypoxia‐inducible factor‐1α (HIF‐1α) (target sequence):

(GCCGCTCAATTTATGAATATTCTCGAGAATATTCATAAATTGAGCGGC) and a non‐targeting scrambled shRNA control (sequence: CCTAAGGTTAAGTCGCCCTCGCTCGAGCGAGGGCGACTTAACCTTAGG) were synthesized. Each shRNA sequence was individually cloned into a vector plasmid containing a cytomegalovirus (CMV) promoter and an enhanced green fluorescent protein (EGFP) reporter gene. The final constructs were designated as AAV‐U6‐HIF‐1α‐shRNA‐CMV‐EGFP and AAV‐U6‐scrambled‐shRNA‐CMV‐EGFP respectively. All plasmid constructs were verified by restriction enzyme digestion and DNA sequencing.

For viral packaging the respective target plasmid was co‐transfected with helper plasmids into HEK293T cells using the calcium phosphate precipitation method. Cells were harvested 72 h post‐transfection. The viral particles were subsequently released by repeated freeze–thaw cycles, precipitated with polyethylene glycol 8000 (PEG‐8000) at a final concentration of 8%, and collected by centrifugation. The crude viral lysate was then purified by caesium chloride (CsCl) density gradient centrifugation, followed by buxffer exchange and concentration using ultrafiltration. The titre of the purified AAV9 preparations was determined by quantitative PCR (qPCR) and adjusted to 1 × 10^12^ genomic copies (GC) per millilitre.

### 2.2 Animal Experiments

All experimental procedures were conducted in accordance with the National Institutes of Health guidelines (Guide for the Care and Use of Laboratory Animals, NIH Publication No. 85–23, revised 1996) and were approved by the Institutional Animal Care and Use Committee of Fudan University. A total of 8–10 mice were initially enrolled per group to account for expected peri‐operative mortality. Only mice that survived the entire experimental protocol and exhibited LVEF < 50% at 1 week post‐MI were included in the final analysis (*n* = 5 per group). Mice that died within 48 h after MI surgery were excluded from subsequent analyses.

Male C57BL/6 mice (6 weeks old) were purchased from Shanghai SLAC Laboratory Animal Co. Ltd. and maintained under standard conditions (12 h light/12 h dark cycle) with free access to food and water. The mice were randomly assigned to five experimental groups (*n* = 5 per group): sham myocardial infarction (MI), MI + Qiliqiangxin (MI + QL), MI + QL + HIF‐1α‐shRNA (MI + QL + HIF‐1α‐shRNA), and MI + QL + scrambled‐shRNA (MI + QL + scrambled‐shRNA) (initial *n* = 8 each, final *n* = 5 each).

To achieve cardiac‐specific knockdown of hypoxia‐inducible factor‐1α (HIF‐1α), mice in the MI + QL + HIF‐1α‐shRNA group received a single tail‐vein injection of 200 μL AAV9 carrying HIF‐1α‐shRNA (1 × 10^12^ GC/ml). Control mice in the MI + QL + scrambled‐shRNA group were injected with an equivalent volume of AAV9 containing a non‐targeting scrambled shRNA. These injections were administered 4 weeks prior to MI induction to allow for sufficient viral transduction and gene knockdown. Preliminary validation confirmed that this protocol resulted in transduction of approximately 70%–80% of myocardial fibres and reduced myocardial HIF‐1α expression by about 70% at 4 weeks post‐injection.

Myocardial infarction was induced Via permanent ligation of the left coronary artery (LCA) using a minimally invasive technique without mechanical ventilation [8]. Briefly mice were anesthetized with 2% isoflurane. A small skin incision was made on the left chest wall. Following dissection of the pectoral muscles the intercostal space between the fourth and fifth ribs was gently opened using a mosquito clamp to penetrate the pleura and pericardium. The heart was exteriorized through this opening. The LCA was identified and ligated approximately 3 mm from its origin with a 6–0 silk suture. The heart was then immediately returned to the thoracic cavity, residual air was manually evacuated, and the muscle and skin layers were closed. Mice in the sham group underwent an identical surgical procedure excluding LCA ligation.

Transthoracic echocardiography was performed 1 week after MI surgery. Only mice exhibiting a left ventricular ejection fraction (LVEF) below 50% were included in the subsequent therapeutic phase of the study. Starting from this time point (1 week post‐MI), mice in the MI + QL, MI + QL + HIF‐1α‐shRNA, and MI + QL + scrambled‐shRNA groups received *QL* via oral gavage at a daily dose of 0.5 g/kg for four consecutive weeks. Mice in the sham and MI groups received an equivalent volume of saline vehicle for the same duration.

### 2.3 Echocardiography

Following 4 weeks of drug administration, transthoracic echocardiography was performed using a Vevo 2100 Imaging System (FUJIFILM VisualSonics Inc., Toronto, Canada). Mice were anesthetized with 2% isoflurane and examined with a 30‐MHz high‐frequency transducer. Two‐dimensional imaging was used to obtain standard parasternal long‐axis and short‐axis views. Key cardiac parameters were measured, including left ventricular internal diameter at diastole (LVIDd) and systole (LVIDs), left ventricular end‐diastolic volume (LVEDV), left ventricular end‐systolic volume (LVESV), left ventricular fractional shortening (LVFS) and left ventricular ejection fraction (LVEF). All measurements were obtained from three consecutive cardiac cycles and averaged. The echocardiographic analysis was conducted by an experienced investigator who was blinded to the group allocations.

### 2.4 Small‐Animal Positron Emission Tomography (PET) Imaging

Following the completion of all interventions, myocardial glucose uptake was assessed using a dedicated small‐animal PET system (Metis PET, Madic, Shandong, China). Mice were anesthetized with 2.0% isoflurane and injected intravenously Via the tail vein with 2‐deoxy‐2‐[^18^F]fluoro‐D‐glucose (^18^F‐FDG) at an approximate dose of 10 μCi per gram of body weight. After a 60‐min uptake period under sustained anaesthesia, an emission scan was performed. During scanning, mice were maintained in a prone position within the scanner aperture under continuous anaesthesia (1.5% isoflurane in pure oxygen delivered at 1.0 L/min Via a nose cone). The total acquisition time was 30 min.

Image reconstruction and analysis were conducted using the manufacturer's software (Metis Viewer Version 1.0 for Windows). A volumetric region of interest was drawn to encompass the left ventricular myocardium and quantify the three‐dimensional distribution of ^18^F‐FDG. Myocardial tracer uptake was expressed as the standardized uptake value (SUV), calculated according to the following formula: SUV = (Radioactivity concentration in the myocardium [Bq/mL]) / (Injected ^18^F‐FDG dose [Bq] / body weight [g]). No additional reference region normalization was applied.

### 2.5 Morphology, Histopathology, Immunohistochemistry, and Oxidative Stress Measurement

At the conclusion of the study, mice were euthanized. Blood was collected Via cardiac puncture, allowed to clot, and centrifuged at 3000 rpm for 20 min to obtain serum, which was stored at −80°C. The heart was rapidly excised, rinsed with cold saline, and weighed to calculate the heart weight‐to‐body weight (HW/BW) ratio. The ventricular tissue between the ligation site and the apex was transversely divided into three portions: one for frozen sections, one for paraffin embedding and the remainder was flash‐frozen in liquid nitrogen and stored at −80°C for subsequent analysis.

For assessment of oxidative stress, frozen tissue samples were embedded in O.C.T. compound (Tissue‐Tek) and sectioned at 4 μm thickness. Sections were incubated with a 10 μmol/L dihydroethidium (DHE) fluorescent probe (Beyotime, Shanghai, China) at 37°C for 30 min in the dark to detect superoxide anion generation. Fluorescence, indicative of intracellular reactive oxygen species (ROS), was visualized and captured using a fluorescence microscope (Leica, Wetzlar, Germany) at 488 nm excitation and 520 nm emission wavelengths. The DHE fluorescence intensity was quantified using ImageJ software (NIH, Bethesda, MD, USA).

For histopathological evaluation, tissue samples were fixed in 4% paraformaldehyde, paraffin‐embedded and sectioned at 4 μm. Sections were stained with Haematoxylin and Eosin (H&E) and Masson's trichrome. Myocardial fibrosis was quantified by calculating the collagen volume fraction (CVF) in the peri‐infarct area as the ratio of the fibrotic (blue‐stained) area to the total left ventricular myocardial area using ImageJ software.

Microvascular density was assessed by CD31 immunohistochemistry. Briefly, deparaffinized sections were incubated with a rabbit anti‐mouse CD31 monoclonal antibody (1:500 dilution; Abcam, Cambridge, UK), followed by appropriate biotinylated secondary antibodies and streptavidin‐biotin‐peroxidase complex (Boster, Wuhan, China). The reaction was visualized using 3,3′‐diaminobenzidine (DAB), with brown staining considered positive. Microvessel density was expressed as the number of CD31‐positive endothelial cell clusters or tubular structures counted in five randomly selected peri‐infarct high‐power fields (400× magnification).

Myocardial apoptosis was detected using a terminal deoxynucleotidyl transferase dUTP nick end labeling (TUNEL) assay kit (Roche, Mannheim, Germany) according to the manufacturer's instructions. Cells with brown‐stained nuclei were considered TUNEL‐positive. The apoptotic index was calculated as the percentage of TUNEL‐positive nuclei relative to the total number of nuclei in five randomly selected peri‐infarct high‐power fields (400×). All images were captured and analysed using a high‐resolution digital image analysis system (Qwin V3, Leica, Germany).

### 2.6 Assessment of Serum NT‐proBNP and LDH

Serum N‐terminal pro‐B‐type natriuretic peptide (NT‐proBNP) concentration was quantified using a commercially available sandwich enzyme‐linked immunosorbent assay (ELISA) kit (Well Biotechnology Co., Shanghai, China), strictly following the manufacturer's protocol. The absorbance was measured at 450 nm using a microplate reader, and sample concentrations were interpolated from a standard curve.

Serum lactate dehydrogenase (LDH) activity was determined with a colorimetric activity assay kit (Sigma‐Aldrich, USA). Briefly, 5 μL of serum sample was added to the reaction mixture in duplicate. The change in absorbance at 450 nm (ΔA450) over a defined period was measured for each sample. LDH activity (nmol/min/mL, equivalent to U/L) was calculated by comparing the ΔA450 of samples to a standard curve, as per the kit's instructions.

### 2.7 Measurement of Myocardial ATP Production, PDH Activity, and CS Activity

Myocardial ATP content was determined using an Enhanced ATP Assay Kit (Beyotime, China) according to the manufacturer's instructions. The measured ATP levels were normalized to the total protein concentration, as quantified by a BCA protein assay kit (Beyotime, China) and expressed as nmol per mg of protein (nmol/mg protein).

The activities of pyruvate dehydrogenase (PDH) and citrate synthase (CS) were assessed using commercial activity assay kits (Sigma‐Aldrich, USA). Approximately 10 mg of myocardial tissue was homogenized in the respective assay buffers provided with each kit and centrifuged. The resulting supernatant was collected for analysis. For each assay, duplicate aliquots of the supernatant were mixed with the corresponding reaction mixtures. The change in absorbance was monitored spectrophotometrically: PDH activity was measured by the change in absorbance at 450 nm (ΔA₄₅₀), and CS activity was measured by the change in absorbance at 412 nm (ΔA₄₁_2_). The activity for each sample was calculated based on a standard curve and normalized to the protein concentration of the supernatant.

### 2.8 Western Blot Analysis

Myocardial tissues were homogenized in radioimmunoprecipitation assay (RIPA) lysis buffer (Beyotime, China) supplemented with protease and phosphatase inhibitor cocktails (Sigma‐Aldrich, USA). The homogenates were centrifuged, and the protein concentration of the supernatant was determined. Equal amounts of protein (20 μg per lane) were separated by electrophoresis on 12% SDS‐polyacrylamide gels and subsequently transferred onto polyvinylidene difluoride (PVDF) membranes (Millipore, USA).

The membranes were blocked with 5% bovine serum albumin (BSA) and then incubated overnight at 4°C with the following primary antibodies (all from Cell Signalling Technology, USA, unless specified): anti‐HIF‐1α (1:1000), anti‐macrophage migration inhibitory factor (MIF; 1:1000), anti‐AMPKα (1:1000), anti‐phospho‐AMPKα (Thr172; 1:1000), anti‐hexokinase 2 (HK2; 1:1000), anti‐pyruvate dehydrogenase (PDH; 1:1000), anti‐citrate synthase (CS; 1:1000) and anti‐glucose transporter 4 (GLUT‐4; 1:1000, Abcam, UK). Following washes, the membranes were incubated with appropriate horseradish peroxidase (HRP)‐conjugated secondary antibodies (1:10000, Abbkine, USA) for 2 h at room temperature.

To control for loading, membranes were probed with an HRP‐conjugated anti‐β‐actin monoclonal antibody (1:10000, Bioworld Technology, USA). Protein bands were visualized using an enhanced chemiluminescence (ECL Plus) substrate (Thermo Scientific, USA) and quantified using Image Lab Software (Bio‐Rad Laboratories, USA). The expression level of each target protein was normalized to that of β‐actin.

### 2.9 Quantitative Real‐Time PCR (qRT‐PCR) Analysis

Total RNAs were isolated from myocardial tissues using the TRIzol RNA extraction kit (Invitrogen, CA, USA) and reverse transcribed into cDNA using a PrimeScript RT reagent kit (Takara, Japan). Quantitative real‐time PCR was performed with the SYBR Premix Ex Taq kit (Takara, Japan) in a 10 μL reaction volume on the CFX Connect Real‐Time System (Bio‐Rad Laboratories, CA, USA). The primers used for PCR were synthesized and provided by Sangon Biotech (Shanghai, China). (Table 1). Relative mRNA expression was normalized to β‐actin as an endogenous control using the standard 2^−ΔΔCt^ method.

**TABLE 1:** Primers sequences used for qRT‐PCR quantification.

Genes	Forward primer	Reverse primer
HIF‐1α	5′‐CTGCCACTGCCACCACAACTG‐3′	5′‐TGCCACTGTATGCTGATGCCTTAG‐3′
MIF	5′‐GCATCGGCAAGATCGGTGGTG‐3′	5′‐ACGTTGGCAGCGTTCATGTCG‐3′
GLUT‐4	5′‐AGCCAGCCTACGCCACCATAG‐3′	5′‐TCCGTCGTCCAGCTCGTTCTAC‐3′
HK2	5′‐GCGTGGATGGCTCTGTCTACAAG‐3′	5′‐GGAGGAAGCGGACATCACAATCG‐3′
PDK4	5′‐GAAGAGCCCAGAAGACCAGAAAGC‐3′	5′‐CCAGGATGCCTTGAGCCATTGTAG‐3′
CS	5′‐GCAGCAGTATCGGAGCCATTGAC‐3′	5′‐TGAACTGAGGGTCGGTGTAGCC‐3′
β‐Actin	5′‐GAAGTGTGACGTTGACATCCG‐3′	5′‐TGCTGATCCACATCTGCTGGA‐3′

Abbreviations: CS, citrate synthase; GLUT‐4, glucose transporter‐4; HIF‐1α, hypoxia‐induced factor‐1α; HK2, hexokinase‐2; MIF, macrophage migration inhibitory factor; PDK4, pyruvate dehydrogenase kinase‐4.

### 2.10 Statistical Analysis

Values are expressed as the mean ± standard deviation (SD). Data between different groups were compared using one‐way ANOVA. *p <* 0.05 was considered statistically significant. All statistical analyses were performed using SPSS software (version 19.0, SPSS Inc. USA).

## 3 Results

### 3.1 Validation of Cardiac AAV9 Transduction and HIF‐1α Knockdown

**FIGURE 1:**
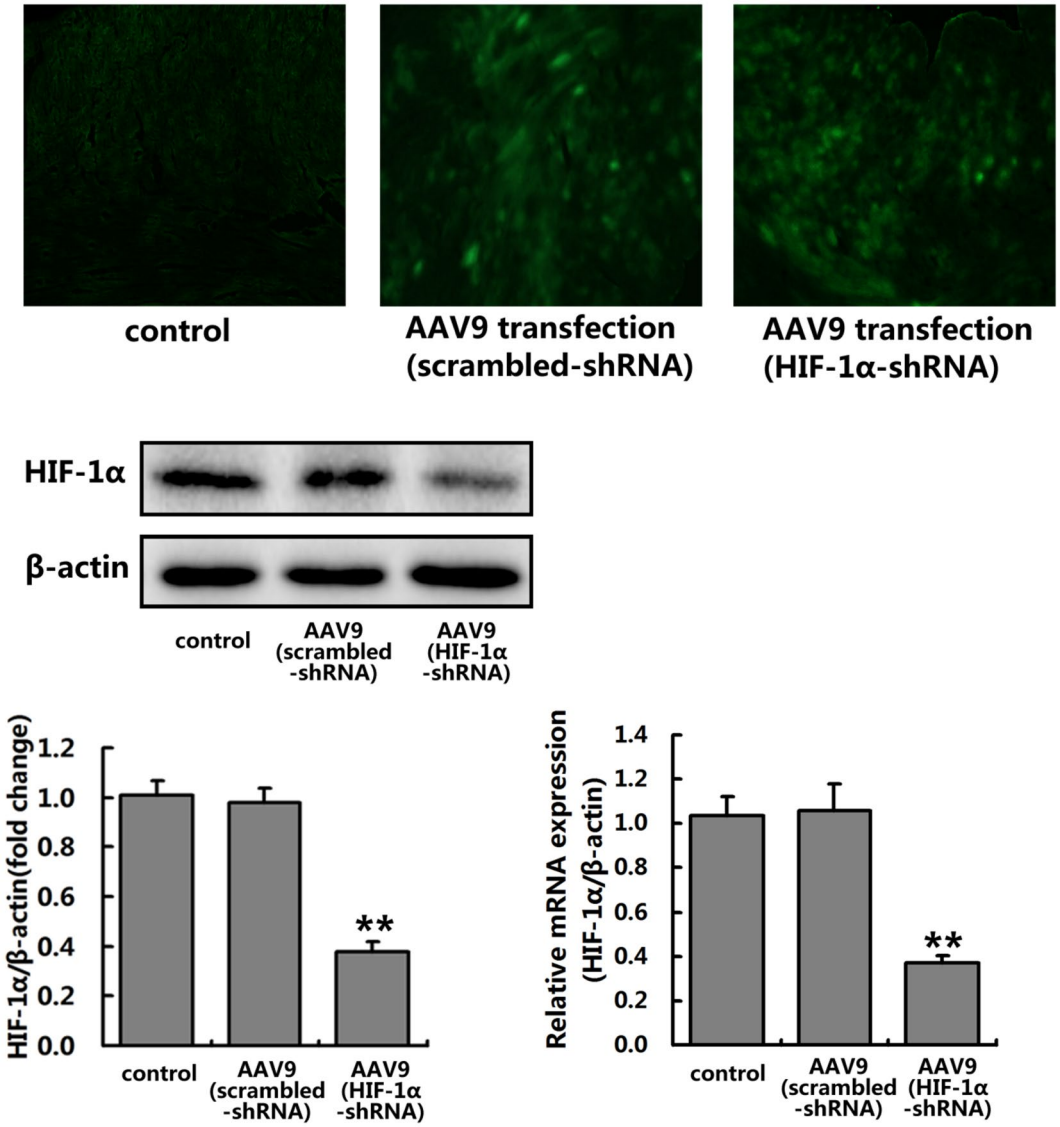
Verification of successful AAV9 cardiac transduction and genetic HIF‐1α knockdown. Green fluorescence was visualized under fluorescence microscopy (100×) after AAV transfection (either scrambled‐shRNA or HIF‐1α‐shRNA). HIF‐1α expression was significantly knocked down in myocardium transfected with AAV‐ HIF‐1α‐shRNA at both the protein and mRNA levels. Compared with the control group, **p < 0.01.

Both AAV9 vectors (encoding HIF‐1α‐shRNA or scrambled‐shRNA) co‐expressed green fluorescent protein (GFP). Fluorescence microscopy analysis performed 4 weeks after tail‐vein injection confirmed robust cardiac transduction, with GFP signal detected in approximately 70%–80% of myocardial fibres. Western blot and quantitative RT‐PCR (qRT‐PCR) analyses demonstrated that myocardial HIF‐1α expression was successfully knocked down. Mice receiving AAV9‐HIF‐1α‐shRNA exhibited a significant reduction in both HIF‐1α protein and mRNA levels (by approximately 70%) compared to controls. In contrast, administration of AAV9‐scrambled‐shRNA had no effect on myocardial HIF‐1α expression (Figure 1).

### 3.2 Qiliqiangxin Attenuates MI‐Induced Cardiac Remodelling and Improves Cardiac Function

Prior to treatment initiation, echocardiographic parameters, including left ventricular internal dimensions and volumes (LVIDd, LVIDs, LVEDV, LVESV) as well as functional indices (LVFS, LVEF), showed no significant differences among the groups subjected to myocardial infarction (MI). After 4 weeks of treatment, mice in the MI group exhibited significant pathological cardiac remodelling and dysfunction compared to the sham group. This was evidenced by increases in the heart weight‐to‐body weight (HW/BW) ratio, left ventricular internal dimensions and volumes (LVIDd, LVIDs, LVEDV, LVESV), and serum NT‐proBNP levels, accompanied by decreases in left ventricular fractional shortening (LVFS) and ejection fraction (LVEF) (all *p* < 0.01).

**FIGURE 2:**
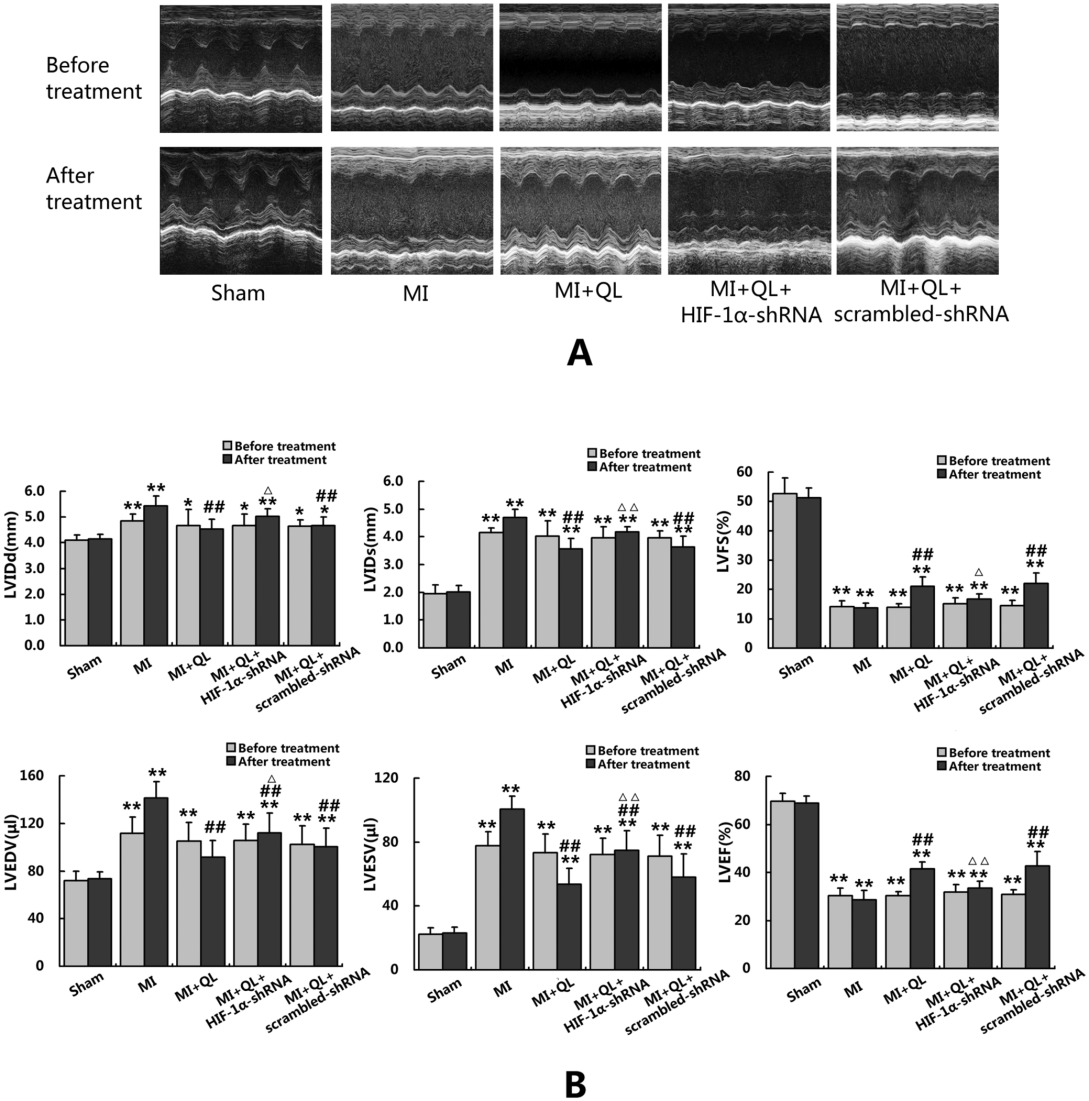
Echocardiographic assessment before and after treatment. A. Typical M‐mode echocardiographic images for each group before and after 4 weeks of drug administration. B. Left ventricular diameter and volume, together with LVFS and LVEF were compared among different groups. LVIDd, left ventricular internal dimension at diastole; LVIDs, left ventricular internal dimension at systole; LVEDV, left ventricular end‐diastolic volume; LVESV, left ventricular end‐systolic volume; LVFS, left ventricular fractional shortening; LVEF, left ventricular ejection fraction; MI, myocardial infarction. Compared with the sham group, *p < 0.05, **p < 0.01; compared with the MI group, ^##^ *p <* 0.01; compared with the MI + *QL* group, ^△^ *p <* 0.05, ^△△^ *p <* 0.01.

**FIGURE 3:**
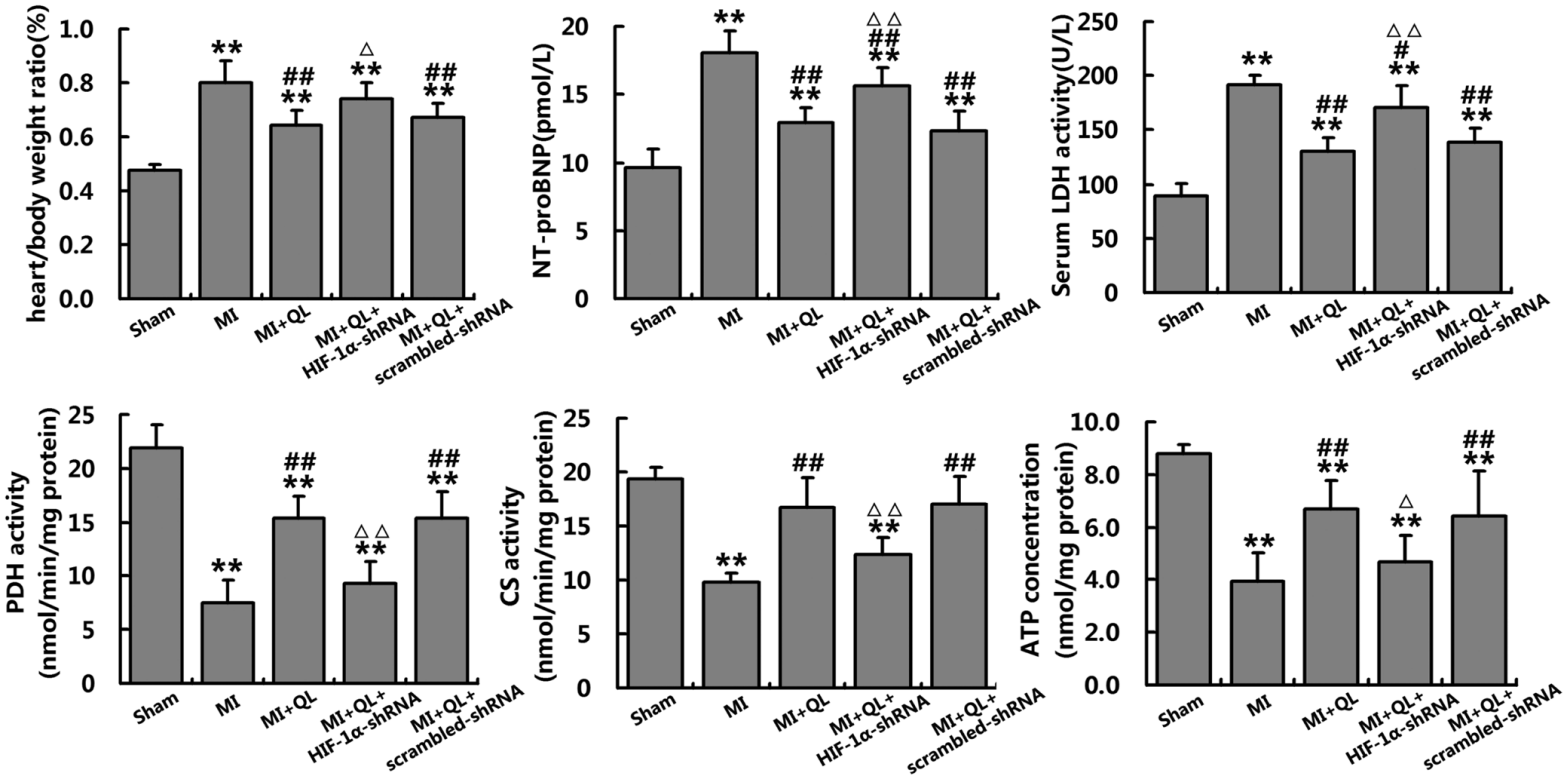
Comparison of heart/body weight ratio, serum NT‐proBNP level and LDH activity, myocardial PDH and CS activity and ATP concentration among different groups. LDH, lactate dehydrogenase; PDH, pyruvate dehydrogenase; CS, citrate synthase. Compared with the sham group, **p < 0.01; compared with the MI group, ^#^ *p <* 0.05; compared with the MI + *QL* group, ^△^ *p <* 0.05, ^△△^ *p <* 0.01.

Treatment with *QL* significantly ameliorated these alterations. The MI + QL group showed a reduced HW/BW ratio, attenuated left ventricular dilation (decreased LVIDd, LVIDs, LVEDV, LVESV), lower serum NT‐proBNP and improved cardiac function (increased LVFS and LVEF) compared to the untreated MI group (*p* < 0.01). Notably, the beneficial effects of *QL* on cardiac structure and function were abolished by prior genetic knockdown of HIF‐1α (MI + QL + HIF‐1α‐shRNA group, *p* < 0.05 vs. MI + QL group), but were preserved in mice receiving the control scrambled shRNA (MI + QL + scrambled‐shRNA group) (Figure 2 and Figure 3).

### 3.3 Qiliqiangxin Enhances Myocardial Glucose Uptake in a HIF‐1α‐Dependent Manner

Small‐animal PET imaging with ^18^F‐FDG provided clear visualization of myocardial glucose uptake. After 4 weeks of intervention, the MI group demonstrated significantly lower myocardial ^18^F‐FDG uptake compared to the sham group (SUVmean: 2.50 ± 0.87 vs. 6.30 ± 0.39, *p* < 0.01).

Treatment with *QL* significantly improved glucose uptake in post‐MI hearts. The MI + QL group exhibited a markedly higher ^18^F‐FDG uptake compared to the untreated MI group (SUVmean: 5.33 ± 0.50 vs. 2.50 ± 0.87, *p* < 0.01). This beneficial effect of QL on enhancing myocardial glucose uptake was attenuated by HIF‐1α knockdown performed 4 weeks prior to the induction of myocardial infarction.

**FIGURE 4:**
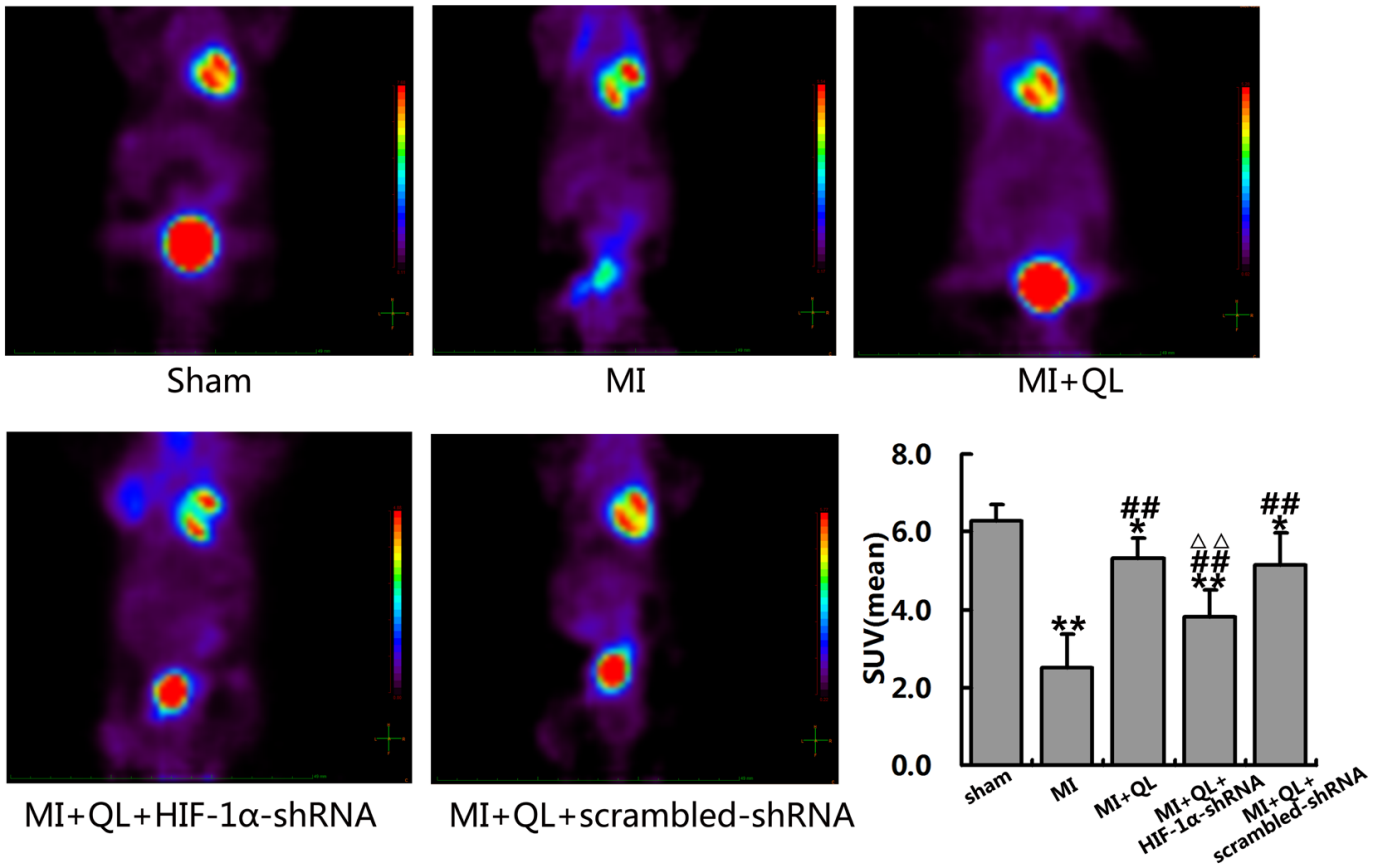
Representative positron emission tomography (PET) images for myocardial ^18^F‐FDG uptake from the five groups of animals. ^18^F‐FDG uptake was calculated and normalized as a standardized uptake value (SUV) Compared with the sham group, *p < 0.05, **p < 0.01; Compared with the MI group, ^##^ *p <* 0.01; Compared with the MI + *QL* group, ^△△^ *p <* 0.01.

The MI + QL + HIF‐1α‐shRNA group showed a lower ^18^F‐FDG uptake compared to the MI + QL group (SUVmean: 3.79 ± 0.71 vs. 5.33 ± 0.50, *p* < 0.01). In contrast, administration of the control scrambled shRNA did not affect the *QL*‐mediated increase in glucose uptake (Figure 4).

### 3.4 Qiliqiangxin Ameliorates Myocardial Fibrosis, Promotes Angiogenesis, Reduces Oxidative Stress, and Inhibits Cardiomyocyte Apoptosis

Histopathological analysis of Haematoxylin and Eosin (H&E)‐stained sections from the infarct border zone revealed substantial structural disruption in the MI group, characterized by disorganized cardiomyocyte arrangement, myofibrillar lysis, and inflammatory cell infiltration. *QL* treatment partially reversed these pathological changes, an effect that was attenuated by HIF‐1α knockdown.

Quantification of myocardial fibrosis by Masson's trichrome staining showed a significantly higher collagen volume fraction (CVF) in the MI group compared to the sham group (18.33% ± 1.36% vs. 1.53% ± 0.50%, *p* < 0.01). *QL* treatment markedly reduced fibrosis (9.66% ± 2.25% vs. 18.33% ± 1.36%, *p* < 0.01). This anti‐fibrotic effect was abolished by HIF‐1α‐shRNA transfection (16.06% ± 2.30% vs. 9.66% ± 2.25%, *p* < 0.01).

Immunohistochemical staining for CD31 demonstrated that *QL* promoted microvascular angiogenesis, as evidenced by a significantly higher microvessel density compared to the MI group (*p* < 0.01). This pro‐angiogenic effect was also abrogated by HIF‐1α knockdown (*p* < 0.01).

Assessment of oxidative stress Via dihydroethidium (DHE) staining revealed a significant increase in reactive oxygen species (ROS) production in the MI group compared to sham controls (fluorescence intensity: 21.04 ± 3.50 vs. 5.48 ± 1.65, *p* < 0.01). *QL* treatment effectively suppressed ROS formation (11.01 ± 2.50 vs. 21.04 ± 3.50, *p* < 0.01), an effect that was attenuated in the HIF‐1α knockdown group (18.56 ± 1.54 vs. 11.01 ± 2.50, *p* < 0.01).

**FIGURE 5:**
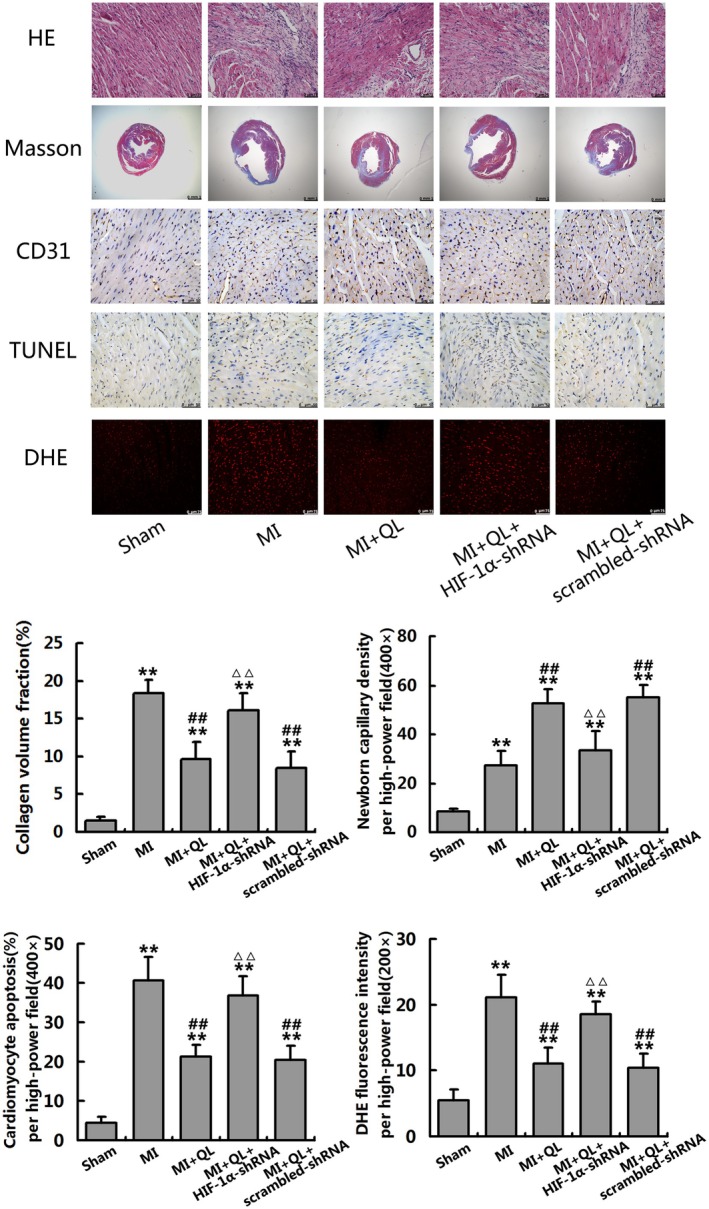
Typical photography of HE staining (200×), Masson's trichrome staining (12.5×), CD31 staining (400×), TUNEL staining (400×), and DHE staining (200×) for assessment of myocardial arrangement, collagen volume fraction (CVF), newborn capillary density, cardiomyocyte apoptosis, and intracellular reactive oxygen species (ROS), respectively. Compared with the sham group, **p < 0.01; Compared with the MI group, ^##^ *p <* 0.01; Compared with the MI + *QL* group, ^△△^ *p <* 0.01.

Finally, TUNEL assay indicated a significantly higher rate of cardiomyocyte apoptosis in the MI group than in the sham group (40.55% ± 5.96% vs. 4.57% ± 1.32%, *p* < 0.01). *QL* treatment significantly reduced apoptosis (21.26% ± 3.03% vs. 40.55% ± 5.96%, *p* < 0.01). This anti‐apoptotic effect was abolished by HIF‐1α‐shRNA transfection (36.73% ± 5.04% vs. 21.26% ± 3.03%, *p* < 0.01) (Figure 5).

### 3.5 Qiliqiangxin Promotes Myocardial Glucose Aerobic Oxidation and ATP Production

To assess the effect of *QL* on glucose aerobic oxidation, we measured key metabolic markers. Compared to the sham group, mice in the MI group exhibited significantly higher serum lactate dehydrogenase (LDH) activity (191.60 ± 9.60 U/L vs. 89.49 ± 11.19 U/L, *p* < 0.01), along with markedly reduced myocardial activity of pyruvate dehydrogenase (PDH) (7.49 ± 2.14 vs. 21.89 ± 2.10 nmol/min/mg protein, *p* < 0.01) and citrate synthase (CS) (9.77 ± 0.92 vs. 19.41 ± 0.91 nmol/min/mg protein, *p* < 0.01), indicating impaired aerobic glucose oxidation post‐MI.

*QL* treatment significantly ameliorated these metabolic disturbances. The MI + QL group showed decreased serum LDH activity (130.74 ± 12.67 U/L vs. 191.60 ± 9.60 U/L, *p* < 0.01) and increased myocardial PDH (15.37 ± 2.09 vs. 7.49 ± 2.14 nmol/min/mg protein, *p* < 0.01) and CS activity (16.67 ± 2.80 vs. 9.77 ± 0.92 nmol/min/mg protein, *p* < 0.01) compared to the untreated MI group. However, this restorative effect of *QL* on enzyme activities was abolished in mice receiving HIF‐1α‐shRNA (*p* < 0.01).

Consistent with impaired oxidative metabolism, myocardial ATP content was significantly lower in the MI group than in the sham group (3.94 ± 1.08 vs. 8.76 ± 0.38 nmol/mg protein, *p* < 0.01). *QL* treatment enhanced ATP production (6.69 ± 1.09 vs. 3.94 ± 1.08 nmol/mg protein, *p* < 0.01). This beneficial effect on ATP synthesis was attenuated by HIF‐1α knockdown (4.69 ± 0.97 vs. 6.69 ± 1.09 nmol/mg protein, *p* < 0.05) (Figure 3).

### 3.6 Qiliqiangxin Upregulates Glucose Metabolism‐Related Enzymes Via the HIF‐1α/MIF/AMPK Axis

First, we assessed the expression of hypoxia‐inducible factor‐1α (HIF‐1α) and macrophage migration inhibitory factor (MIF) in myocardial tissue. *QL* treatment significantly increased the expression of both HIF‐1α and MIF at the mRNA and protein levels compared to the untreated MI group.

Concomitantly, the protein level of phospho‐AMPKα^(Thr172)^ (p‐AMPKα), reflecting AMP‐activated protein kinase (AMPK) activation, was significantly upregulated in the MI + QL group.

We further evaluated the expression of key proteins involved in glucose transport and metabolism. Myocardial expression of glucose transporter type 4 (GLUT‐4), hexokinase 2 (HK2), pyruvate dehydrogenase (PDH), and citrate synthase (CS) was significantly downregulated in the MI group. *QL* treatment markedly increased the expression of these proteins (*p* < 0.01). Conversely, the mRNA expression of pyruvate dehydrogenase kinase 4 (PDK4), an inhibitor of the PDH complex, was upregulated in the MI group and significantly downregulated by QL treatment (*p* < 0.01).

**FIGURE 6:**
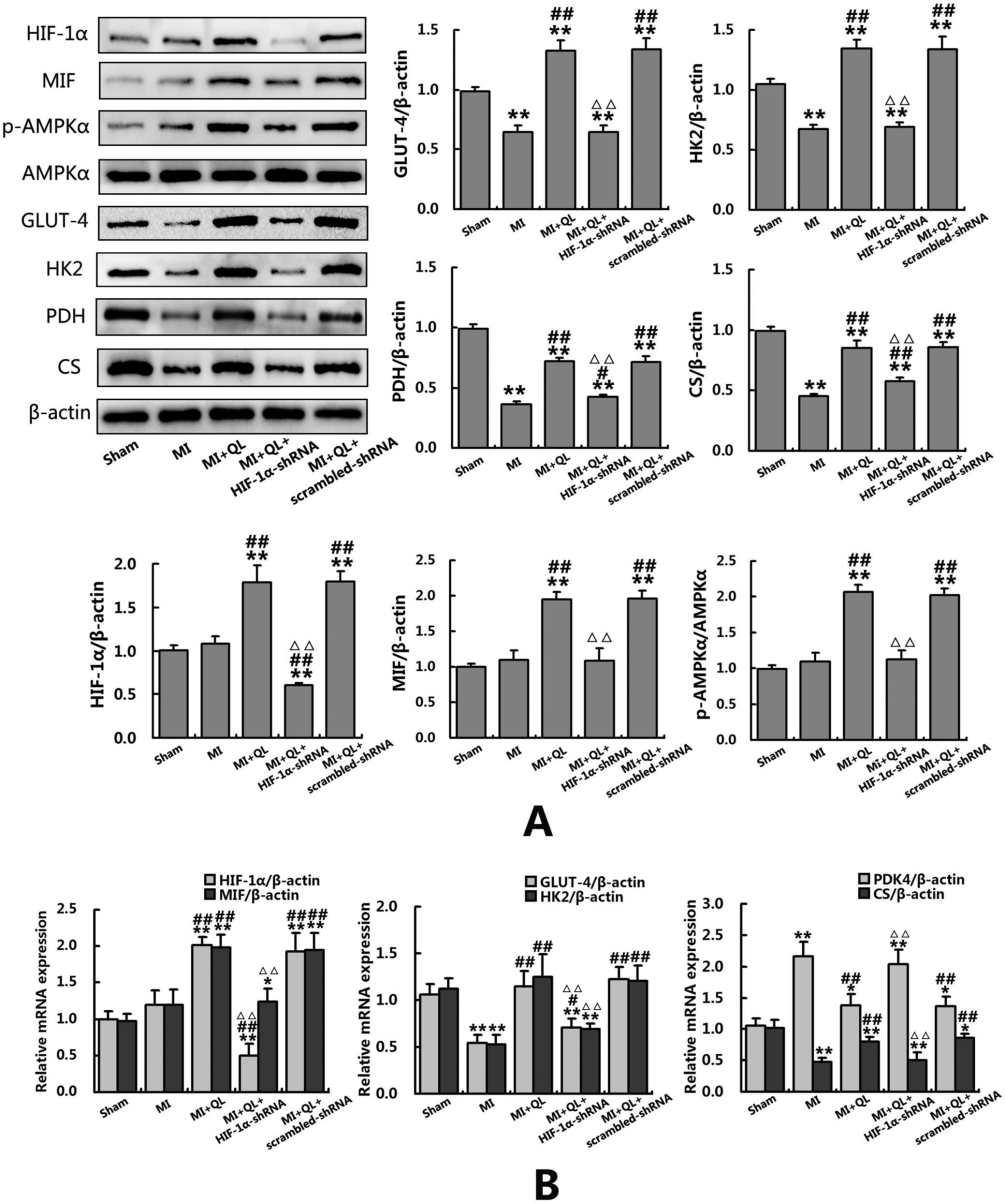
Protein and mRNA expressions of peri‐infarct myocardium among experimental animals. A. Relative protein expressions including HIF‐1α, MIF, p‐AMPKα, GLUT‐4, HK2, PDH, and CS among the five groups by Western blot. B. Relative mRNA expression, including HIF‐1α, MIF, GLUT‐4, HK2, PDK4 and CS among the five groups by qRT‐PCR. HIF‐1α, hypoxia induced factor‐1α; MIF, macrophage migration inhibitory factor; AMPK, AMP‐activated protein kinase; GLUT‐4, glucose transporter‐4; HK2, hexokinase‐2; PDH, pyruvate dehydrogenase; PDK4, pyruvate dehydrogenase kinase‐4; CS, citrate synthase. Compared with the sham group, *p < 0.05, **p < 0.01; Compared with the MI group, ^#^ *p <* 0.05, ^##^ *P <* 0.01; Compared with the MI + *QL* group, ^△△^ *p <* 0.01.

All the aforementioned regulatory effects of *QL*—including the upregulation of MIF, activation of AMPK (p‐AMPKα), and modulation of metabolic enzymes (GLUT‐4, HK2, PDH, CS, PDK4)—were blunted in mice subjected to HIF‐1α knockdown (MI + QL + HIF‐1α‐shRNA group, *p* < 0.01) (Figure 6). These results suggest that QL modulates cardiac glucose metabolism post‐MI by regulating key enzymes, a process in which HIF‐1α signalling and subsequent MIF expression and AMPK activation appear to play critical roles.

## 4 Discussion

(The main findings of the present study are: 1) *QL* promoted myocardial glucose uptake and aerobic oxidation, contributing to attenuated cardiac remodelling in a murine model of(post‐MI heart failure; 2) genetic knockdown of HIF‐1α abolished these beneficial effects, suggesting the involvement of the HIF‐1α/MIF/AMPK signalling axis.

Angiogenesis is pivotal in the pathophysiology of MI and heart failure and is closely linked to energy metabolism. Enhancing functional coronary vasculature can reprogram myocardial metabolic pathways and confer protection against ischemic injury. Our prior work demonstrated that *QL* protects the myocardium from ischemic damage by activating HIF‐1α expression [9, 10]. Under acute hypoxia, HIF‐1α upregulates key glycolytic enzymes, facilitating cellular adaptation to acute stress [11]. In the context of chronic hypoxia, sustained HIF‐1α activation promotes angiogenesis, thereby improving blood and oxygen supply, which may in turn support mitochondrial respiration and aerobic metabolism. Our current results indicate that *QL* reduced ROS production, enhanced myocardial glucose uptake, and improved aerobic oxidation in an HIF‐1α‐dependent manner, effects potentially attributable to its promotion of microvascular angiogenesis.

To further explore the molecular mechanisms underlying *QL*'s regulation of glucose metabolism we examined the expression of macrophage migration inhibitory factor (MIF) and phospho‐AMPKα (p‐AMPKα). *QL* administration increased the expression of both MIF and p‐AMPKα coinciding with HIF‐1α upregulation. Conversely genetic knockdown of HIF‐1α suppressed the expression of MIF and p‐AMPKα. MIF is a pleiotropic cytokine implicated in inflammatory responses as well as in myocardial ischaemia and reperfusion injury. Elevated plasma MIF levels, observed in patients during the early phase of MI may represent a cardioprotective response [12]. Increased MIF levels have also been documented in conditions such as metabolic syndrome, diabetes and chronic kidney disease which are common comorbidities in MI patients [13]. Hypoxia stimulates MIF secretion and its expression is directly regulated by the transcription factor HIF‐1α a master regulator of hypoxic and ischaemic signalling [14]. The HIF‐1α/MIF axis has been investigated in various experimental models, including cardiomyocyte and vascular smooth muscle cell cultures under hypoxic or ischaemia/reperfusion conditions [15, 16].

MIF confers cardioprotection through multiple mechanisms, including activation of AMPK signalling, suppression of apoptotic pathways and attenuation of oxidative stress in the post‐ischemic heart [17]. AMPK activation is integral to metabolic regulation, promoting glucose uptake and mitochondrial oxidative metabolism, including glucose oxidation, fatty acid β‐oxidation and oxidative phosphorylation [18]. By shifting metabolism from anabolic to catabolic pathways, AMPK helps maintain cellular ATP levels through enhanced glucose uptake, glycolysis and oxidative metabolism [19]. Specifically, AMPK activation stimulates GLUT4 translocation to the plasma membrane, thereby increasing glucose uptake. Moreover, elevated p‐AMPK expression protects the heart against ischemic injury by supporting both glycolysis and oxidative phosphorylation to sustain ATP production [20]. In our study, AMPK activation appeared to mediate increased myocardial glucose uptake, enhanced glycolytic flux Via upregulation of HK2, and elevated expression of aerobic oxidation enzymes such as PDH and CS. Concomitant downregulation of PDK4 further promoted PDH complex activity, facilitating the conversion of pyruvate to acetyl‐CoA. Collectively, these adaptations likely contributed to reduced oxidative stress and cardiomyocyte apoptosis, thereby protecting the heart from ischemic injury.

It is well established that under hypoxic conditions HIF‐1α rapidly accumulates dimerizes with its β subunit, and acts as a transcriptional activator for numerous genes involved in glycolysis often shifting energy production from oxidative phosphorylation towards glycolysis particularly in cancer cells. However in non‐cancer cells glucose metabolism adapts flexibly to oxygen availability a phenomenon known as the Pasteur effect [21]. This metabolic flexibility allows cells to utilize various nutrients to survive in a heterogeneous microenvironment exhibiting stable states such as HIF‐1α^high^/pAMPK^low^ HIF‐1α^low^/pAMPK^high^ and HIF‐1α^high^/pAMPK^high^ [19]. In the present study *QL* enhanced myocardial glucose uptake and aerobic oxidation in a post‐MI mouse model in an HIF‐1α‐dependent manner. Although HIF‐1α is known to promote glycolysis by upregulating glycolytic enzymes and glucose transporters it also stimulates microvascular angiogenesis thereby improving perfusion and oxygen supply which may ultimately support aerobic metabolism in the chronically ischemic heart. Notably while HIF‐1α expression in untreated MI mice peaked at 1 week and declined by 4 weeks post‐MI QL treatment sustained significantly higher HIF‐1α levels throughout the four‐week intervention consistent with previous reports of HIF‐1α downregulation in chronic failure [22]. This sustained expression may be attributable to QL‐induced microvascular angiogenesis [9], which improves tissue oxygenation and reduces chronic oxidative stress thereby creating a permissive environment for prolonged HIF‐1α activity. Furthermore our findings suggest that the HIF‐1α/MIF/AMPK axis plays a critical role in mediating the metabolic effects of *QL* as HIF‐1α knockdown abolished its benefits and concurrently downregulated MIF and p‐AMPKα both of which are closely linked to aerobic metabolic pathways.

Improving cardiac energy metabolism after MI is closely associated with reduced oxidative stress and attenuation of cardiomyocyte apoptosis. In this study *QL* acted as a metabolic modulator targeting both substrate utilization and oxidative phosphorylation, offering a potential therapeutic strategy for MI and heart failure. Nevertheless (this study has several limitations: 1) We used cardiac‐specific AAV9‐mediated HIF‐1α knockdown rather than global genetic knockout mice, as complete HIF‐1α knockout is often lethal. (Nonetheless efficient AAV9 transduction allowed for relevant mechanistic insight; 2) While we assessed glucose aerobic metabolism through enzyme expression and activity functional metabolic studies such as flux analysis were not performed and warrant further investigation.

## 5 Conclusion

In summary our study demonstrates that *QL* enhances myocardial glucose uptake and aerobic oxidation in a murine model of post‐MI heart failure. This metabolic improvement was associated with increased ATP production reduced oxidative stress, attenuated cardiomyocyte apoptosis and fibrosis ultimately contributing to favourable cardiac reverse remodelling. The beneficial effects of *QL* appear to be mediated at least in part by HIF‐1α‐dependent microvascular angiogenesis and activation of the HIF‐1α/MIF/AMPK signalling axis. These findings highlight *QL* as a potential therapeutic strategy targeting cardiac energetics in ischemic heart failure.

## Author Contributions

**Chaofu Li:** writing – original draft, writing – review and editing, methodology, validation, visualization. **Zimu Wang:** conceptualization, methodology, software, data curation, writing – original draft, writing – review and editing, funding acquisition, visualization, project administration, supervision. **Yanyan Wang:** conceptualization, investigation, funding acquisition, writing – original draft, writing – review and editing, software, formal analysis, project administration. **Ya'nan Qu:** writing – original draft, writing – review and editing, investigation, funding acquisition, supervision, data curation, project administration, resources. **Zhonglei Xie:** conceptualization, methodology, software, data curation, writing – review and editing, writing – original draft, validation. **Yu Song:** methodology, validation, visualization, writing – review and editing, writing – original draft, conceptualization, investigation, funding acquisition. **Hanqing Zhao:** funding acquisition, visualization, project administration, resources, software, formal analysis, data curation, investigation. **Jingmin Zhou:** data curation, supervision, resources, writing – review and editing, writing – original draft, investigation, validation, formal analysis. **Jingfeng Wang:** writing – original draft, writing – review and editing, visualization, project administration, resources, software, formal analysis, supervision, data curation.

## Funding

This work was supported by General Program of Natural Science Foundation of Shanghai, 24ZR1461400.

National Natural Science Foundation of China, 81870177.

Shanghai Municipal Health and Family Planning Commission, No. 2018JP002.

## Ethics Statement

This study was conducted under the guidelines on humane use and care of laboratory animals for biomedical research published by the National Institutes of Health (No. 85–23, revised 1996). All animal experiments were approved by the Institutional Animal Care and Use Committee of Fudan University.

## Conflicts of Interest

The authors declare no conflicts of interest.

## Data Availability

The data that support the findings of this study are available from the corresponding author upon reasonable request.
